# Clinical Pharmacogenetics Implementation Consortium Guideline for *NAT2* Genotype and Hydralazine Therapy

**DOI:** 10.1002/cpt.70071

**Published:** 2025-09-19

**Authors:** Michael T. Eadon, David W. Hein, Michael A. Andersen, Arlene B. Chapman, Rhonda M. Cooper‐DeHoff, Zeruesenay Desta, Julio D. Duarte, Amanda L. Elchynski, Andrea Gaedigk, Seth E. Karol, Nita A. Limdi, Christelle Lteif, Laura Sosinski, Pablo Zubiaur, Michelle Whirl‐Carrillo, Teri E. Klein, Kelly E. Caudle, Roseann S. Donnelly, José A.G. Agúndez

**Affiliations:** ^1^ Division of Clinical Pharmacology Indiana University School of Medicine Indianapolis Indiana USA; ^2^ Department of Pharmacology and Toxicology University of Louisville School of Medicine Louisville Kentucky USA; ^3^ Department of Clinical Pharmacology Copenhagen University Hospital – Bispebjerg and Frederiksberg Copenhagen Denmark; ^4^ Department of Medicine University of Chicago Chicago Illinois USA; ^5^ Department of Pharmacotherapy and Translational Research and Center for Pharmacogenomics and Precision Medicine University of Florida College of Pharmacy Gainesville Florida USA; ^6^ Department of Pharmacy Arkansas Children’s Hospital Little Rock Arkansas USA; ^7^ Division of Clinical Pharmacology, Toxicology & Therapeutic Innovation, Children’s Mercy Research Institute and School of Medicine University of Missouri‐Kansas City Kansas City Missouri USA; ^8^ Department of Oncology St. Jude Children’s Research Hospital Memphis Tennessee USA; ^9^ Department of Neurology, Heersink School of Medicine University of Alabama at Birmingham Birmingham Alabama USA; ^10^ Walgreens Specialty Indianapolis Indiana USA; ^11^ Department of Clinical Pharmacology Hospital Universitario de la Princesa, Instituto Teófilo Hernando, Universidad Autónoma de Madrid (UAM), Instituto de Investigación Sanitaria La Princesa (IP) Madrid Spain; ^12^ Department of Biomedical Data Science Stanford University Stanford California USA; ^13^ Department of Pharmacy and Pharmaceutical Sciences St. Jude Children’s Research Hospital Memphis Tennessee USA; ^14^ Department of Pharmacy Practice Massachusetts College of Pharmacy and Health Sciences Boston Massachusetts USA; ^15^ Institute of Molecular Pathology Biomarkers Universidad de Extremadura Cáceres Spain

## Abstract

Hydralazine is a vasodilator typically used in the treatment of resistant hypertension and heart failure. N‐acetyltransferase 2 (NAT2) catalyzes the metabolism of hydralazine into inactive metabolites. NAT2 poor metabolizers (historically referred to as “slow acetylators”) are predicted to have increased plasma hydralazine concentrations compared with NAT2 rapid and intermediate metabolizers (historically referred to as “rapid acetylators” and “intermediate acetylators,” respectively), which may lead to both increased clinical efficacy and adverse effects, including drug‐induced systemic lupus erythematosus. This guideline summarizes the evidence from the literature relevant to *NAT2*/hydralazine and provides recommendations for hydralazine prescribing based on *NAT2* genotype‐predicted acetylator phenotype (updates at www.cpicpgx.org).

Hydralazine is a vasodilator used in the treatment of resistant hypertension and heart failure.^1^, ^2^, ^3^ Hydralazine undergoes metabolism via the N‐acetyltransferase 2 (NAT2) enzyme into inactive metabolites. Substantial interindividual variability underlies the pharmacokinetics of hydralazine because the *NAT2* gene is highly polymorphic. The association between *NAT2* genetic variability and hydralazine pharmacokinetics and response is one of the earliest explored in pharmacogenomics.^4^, ^5^, ^6^ Approximately half the world’s population is poor metabolizers (slow acetylators) and half are rapid or intermediate metabolizers (rapid or intermediate acetylators), with significant differences in allele distribution across genetic ancestral populations.^7^ *NAT2*‐poor metabolizer genotypes have been associated with increased circulating hydralazine concentrations, with subsequent effects on antihypertensive response, vasodilation, tachycardia, and potentially drug‐induced systemic lupus erythematosus (SLE).^8^ Hydralazine is still prescribed with reasonable frequency in the setting of resistant hypertension and heart failure; one center reported that 13% of all patients in the kidney clinic were prescribed hydralazine, including 25% of individuals with African ancestry.^8^ The purpose of this guideline is to provide clinicians information that will allow the interpretation of clinical *NAT2* genotype test results so that they can be used to guide hydralazine prescribing. Detailed guidelines for the use of hydralazine, as well as the cost‐effectiveness of *NAT2* genotyping, are beyond the scope of this document. CPIC guidelines are periodically updated at www.cpicpgx.org/guidelines/.

## FOCUSED LITERATURE REVIEW

A systematic literature review focused on *NAT2* genotype and hydralazine pharmacokinetics, efficacy, and toxicity was conducted (see **Supplement, Literature Review**). The evidence is summarized in **Table** **S1**.

## GENE: *NAT2*

### Background

Most clinical laboratories report *NAT2* genotype results using star (*) allele nomenclature. The *NAT2* gene is highly polymorphic, with approximately 50 allele haplotypes defined to date by the Pharmacogene Variation Consortium (PharmVar) (https://www.pharmvar.org/gene/NAT2; see ***NAT2* Allele Definition Table** online).^9^, ^10^, ^11^ PharmVar recently applied its standard allele nomenclature criteria to *NAT2* haplotypes upon transfer to the PharmVar database, which resulted in the reclassification of several *NAT2* alleles to new star allele designations. For example, the legacy *12 allele was reclassified as the new *1 reference allele due to its presence in the hg38 reference genome. Until this reclassification, the *4 allele had been considered the reference allele. Both the *4 and new *1 alleles possess fully functioning enzyme activity. In addition, new star alleles were introduced and others retired. Readers can refer to the GeneFocus review (in preparation) and the *NAT2* page of the PharmVar website for more information (https://www.pharmvar.org/gene/NAT2), including a table that crosswalks between legacy star allele and new PharmVar star allele nomenclature.

The frequencies of these star (*) alleles significantly differ across ancestrally diverse populations.^7^ Alleles are categorized into functional groups as follows: increased function (historically known as “rapid acetylator alleles,” i.e., NAT2*1 and *4), decreased function (historically known as “slow acetylator alleles,” e.g., NAT2*5, *6, *7, and *14), uncertain function (e.g., NAT2*10, *27, *44, *51), and unknown function (e.g., NAT2*57 and *65). Individuals of East Asian ancestry have the highest frequency of increased function alleles, while a greater proportion of individuals of African or European ancestry possess decreased function alleles. The clinical allele function, as described in the ***NAT2* Allele Functionality Table**, was determined based on reported *in vitro* and/or *in vivo* evidence when available.^10^, ^11^ Although there is evidence that alleles convey varying degrees of decreased activity (e.g., the NAT2*6 allele has less activity than the *5 allele^12^, ^13^, ^14^), the CPIC *NAT2* Pharmacogene Curation Expert Panel agreed that there is insufficient clinical evidence to support clinical functional differentiation between decreased function alleles. The committee also made a deliberate decision to classify allele function as increased, decreased, uncertain, or unknown, without a “normal” category, as discussed in the “Other Considerations” section below.

### Genetic test interpretation

The combination of inherited alleles determines a person’s diplotype (also referred to as genotype). **Table** **1** defines each predicted phenotype based on allele function combinations and provides example diplotypes. NAT2 rapid metabolizers (RMs) are characterized by the presence of two increased function alleles (e.g., NAT2*1/*1), resulting in fully functional enzyme activity. NAT2 intermediate metabolizers (IMs) are characterized by the presence of one increased function allele and one decreased function allele (e.g., NAT2*1/*5). NAT2‐poor metabolizers (PMs) are characterized by the presence of two decreased function alleles (e.g., NAT2*5/*5). The “indeterminate” phenotype is assigned when an individual carries one or two uncertain or unknown function alleles. See the ***NAT2* Diplotype–Phenotype Table** online for a complete list of possible diplotypes and the corresponding predicted metabolizer phenotype assignments.^10^, ^11^

**Table 1:** Assignment of predicted NAT2 phenotype based on genotype

Predicted phenotype	Acetylator phenotype (legacy terms)	Genotypes	Examples of *NAT2* diplotypes^a^
NAT2 rapid metabolizer (RM)	Rapid (or fast) acetylator	An individual carrying two increased function alleles	*1/*1, *1/*4, *4/*4
NAT2 intermediate metabolizer (IM)	Intermediate acetylator	An individual carrying one increased function allele and one decreased function allele	*1/*5, *4/*6, *1/*7, *1/*14
NAT2 poor metabolizer (PM)	Slow acetylator	An individual carrying two decreased function alleles	*5/*5, *5/*6, *7/*7, *6/*14
NAT2 indeterminate	n/a	An individual carrying one or two uncertain or unknown function alleles	*10/*27, *5/*51, *7/*44

^a^ Please refer to the ***NAT2* Diplotype–Phenotype Table** online for a complete list. For allele function, please refer to the ***NAT2* Allele Functionality Table**.^10^, ^11^

Tables on the CPIC website contain a list of *NAT2* alleles, the combinations of variants that define each allele, allele function, and reported allele frequencies across major ancestral populations.^10^, ^11^ Many *NAT2* alleles are rare or have only been observed in some population(s). Additionally, many *NAT2* alleles are comprised of various combinations of five single nucleotide variants (SNVs) used to characterize the most common alleles (*1, *4, *5, *6, *7, *14, and *16), which define NAT2 metabolizer status.^15^ A four SNV model defining the common alleles has been shown to have a high NAT2 phenotyping concordance (98.4%).^15^ These SNVs include rs1801280 (c.341T>C for *5 or *16), rs1799930 (c.590G>A for *6), rs1799931 (c.857G>A for *7), and rs1801279 (c.191G>A for *14). With the reclassification of the reference allele from *4 to *1, rs1208 (c.803A>G) is needed to distinguish certain alleles such as the *1 and *4 alleles or *5 and *16 alleles, although no phenotypic difference is expected from rs1208.

### Available genetic test options

See the Genetic Testing Registry (www.ncbi.nlm.nih.gov/gtr/) for more information on commercially available clinical testing options.

### Incidental findings

No inherited diseases or conditions have been consistently or strongly linked to germline genetic variants in *NAT2* independent of xenobiotic metabolism and response. Because NAT2 is involved in the metabolism of carcinogenic compounds, individuals with reduced NAT2 metabolism may have an increased risk of certain cancers, such as bladder cancer^16^ and lung cancer,^17^, ^18^ but these risks are dependent upon the extent of carcinogen exposure. The CPIC writing group does not endorse the utility of *NAT2* genotyping to reliably inform cancer risk. Recent studies suggest that human *NAT2* is a novel genetic factor that influences plasma lipid and cholesterol levels and alters the risk of cardiometabolic disorders.^19^ However, the American College of Medical Genetics and Genomics does not currently recommend return of secondary findings for *NAT2* from exome or genome sequencing.^20^

### Other considerations

The CPIC community held forums and conducted surveys to address the naming conventions for *NAT2* allele function and metabolizer phenotypes. The community decided not to assign any allele as a “normal function” allele. Allele functions were limited to increased, decreased, uncertain, or unknown functional classifications. Likewise, no “normal metabolizer” phenotype category was assigned; phenotypes were limited to rapid, intermediate, poor, or indeterminate metabolizer statuses. The body of NAT2 literature (approximately 4,000 publications) has largely employed the terms rapid/slow acetylator and rapid/slow allele function for decades. The use of the CPIC rapid metabolizer phenotype, rather than normal metabolizer, is an easily recognizable substitute for the rapid acetylator phenotype, acknowledging that this phenotype will be defined by two increased function alleles instead of one increased and one normal function allele (as is the case for other drug‐metabolizing genes, such as *CYP2C19*). The poor metabolizer phenotype held a similar connotation to slow acetylator and was adopted.

NAT2 metabolizer status is divided evenly between rapid/intermediate metabolism and poor metabolism across the global population, yet it is tightly tied to genetic ancestry. Consequently, the writing committee, with input from the broader CPIC membership, ultimately resolved that neither metabolizer phenotype would be classified as normal due to the even global split and extensive use of the historical terms that do not include the term “normal.” In addition, because all phenotypes are actionable for hydralazine prescribing, future clinical decision support may alert providers for rapid or intermediate metabolizers prescribed low doses and for slow metabolizers prescribed high doses. Thus, we identified reasonable implications for implementation, which would warrant rapid, intermediate, and poor metabolizer phenotypes. In summary, the terms rapid, intermediate, and poor metabolizer replaced rapid, intermediate, and slow acetylator, respectively, in this guideline (**Table** **1**).

## DRUG: HYDRALAZINE

### Background

Hydralazine is metabolized into inactive metabolites by NAT2. NAT2 directly acetylates hydralazine, which is then cyclized to form 3‐methyl‐1,2,4‐triazolo‐[3,4a] phthalazine (MTP).^5^ It also acetylates the oxidized metabolite 1‐hydrazinophthalazine (HPZ) into NAc‐HPZ, N‐acetyl‐hydrazine‐phthalazinone.^8^

Hydralazine is indicated for use in resistant hypertension and in afterload reduction for heart failure with reduced ejection fraction, often in conjunction with isosorbide dinitrate.^1^ The drug is also sometimes used in the treatment of hypertensive emergency or hypertension in pregnancy.^21^ The US Food and Drug Administration (FDA) labels for oral hydralazine hydrochloride and BiDil® combination therapy (isosorbide dinitrate and hydralazine) both convey that acetylation status is a predictor of plasma drug concentrations.^22^, ^23^ The FDA label states, “Hydralazine is subject to polymorphic acetylation; slow acetylators generally have higher plasma levels of hydralazine and require lower doses to maintain control of blood pressure.” In the FDA Table of Pharmacogenetic Associations, NAT2 enzyme activity is deemed a predictor of hydralazine systemic concentration.^24^ The FDA drug label for hydralazine contains approved total oral daily doses ranging from 40 mg (10 mg four times daily) to 300 mg (100 mg three times daily). The FDA drug label for BiDil (isosorbide dinitrate and hydralazine hydrochloride) supports a thrice‐daily dosing schedule. Herein, there is potential utility in dose adjustment and titration based on predicted acetylation status. Typical total daily adult starting doses range from 40 to 75 mg, but clinicians may increase starting doses in the setting of severe uncontrolled hypertension or reduce dosing regimens to twice‐daily (off label) to encourage adherence.

The common adverse drug effects (ADEs) of hydralazine include hypotension and tachycardia, which are expected. An uncommon but serious ADE of hydralazine is drug‐induced SLE, often defined serologically by hypocomplementemia and anti‐histone antibody positivity. This ADE is reported to be dose‐dependent, with total daily doses often exceeding 200 mg, particularly in NAT2 PMs or individuals with certain HLA‐DRw4 genotypes.^25^, ^26^, ^27^, ^28^, ^29^, ^30^, ^31^ Cumulative lifetime dose is a risk factor for drug‐induced SLE; patients with exposure to hydralazine doses of ≥400 mg/day for more than 20 years are at increased risk.^31^ Based on the published literature, the American Heart Association guidelines currently recommend limiting total daily hydralazine doses for all patients to 200 mg to avoid drug‐induced SLE^32^ even though the maximum total daily dose recommended by the US FDA is 300 mg.^22^

### Linking genetic variability to variability in drug‐related phenotypes

Both the circulating hydralazine concentrations and blood pressure responses vary in patients prescribed hydralazine. NAT2 polymorphic acetylation affects the pharmacokinetics of oral administration of hydralazine, not intravenous administration, because no first‐pass effect occurs after intravenous administration of hydralazine.^33^ Most studies exploring the relationship between *NAT2* genetic variation and oral hydralazine involve phenotyping, as they were conducted before genotyping technologies were readily available for scientific research. The overall concordance of *NAT2* genotype to NAT2 enzymatic activity phenotype is greater than 90% (**Table** **S1**).^8^, ^12^, ^15^, ^34^, ^35^, ^36^ Although some data suggest the presence of a NAT2 IM phenotype, most studies using phenotyping classified patients into one of two groups—“rapid acetylators” (i.e., NAT2 RMs plus IMs) and “slow acetylators” (i.e., NAT2 PMs).^37^, ^38^, ^39^, ^40^ Therefore, clinical recommendations currently follow the literature, with identical recommendations provided for NAT2 RMs and IMs.

Studies linking *NAT2* genotype with variability in hydralazine pharmacokinetics, efficacy, and toxicity are summarized in **Table** **S1**. The studies primarily focused on the use of hydralazine to treat resistant hypertension. It is this body of evidence that provides the basis for genotype‐predicted NAT2 phenotype‐based therapeutic recommendations when considering treatment with hydralazine (**Table** **2**). With respect to hydralazine pharmacokinetics, studies demonstrate that NAT2 RMs and IMs require a relative dose increase of between 40% and 125% compared to PMs for similar hydralazine exposure.^21^, ^37^, ^41^, ^42^, ^43^, ^44^ For example, hydralazine dosed at 182 mg in NAT2 RMs and 83 mg in NAT2 PMs (119% dose increase) resulted in similar hydralazine exposure.^37^ With respect to hydralazine clinical effect, studies demonstrate that hydralazine is a more effective antihypertensive in NAT2 PMs compared to NAT2 RMs at the same dose and that a higher dose of hydralazine is required in NAT2 RMs to achieve an antihypertensive effect equivalent to NAT2 PMs.^41^, ^43^, ^44^

**Table 2:** Hydralazine oral dosing recommendations based on NAT2 phenotype in adults with resistant hypertension

NAT2 phenotype^a^	Implications for phenotypic measures	Therapeutic recommendation	Classification of recommendation^b^
NAT2 rapid metabolizer (RM)	Predicted to have reduced plasma concentrations and efficacy of hydralazine compared to NAT2 poor metabolizers	Consider a starting total daily dose of at least 75 mg. Titrate up to 300 mg total daily hydralazine dose as tolerated. NAT2 rapid metabolizers typically require a 50–100% higher maintenance dose compared to poor metabolizers	Moderate
NAT2 intermediate metabolizer (IM)	Predicted to have reduced plasma concentrations and efficacy of hydralazine compared to NAT2 poor metabolizers	Consider a starting total daily dose of at least 75 mg. Titrate up to 300 mg total daily hydralazine dose as tolerated. NAT2 intermediate metabolizers typically require a 50–100% higher maintenance dose compared to poor metabolizers	Moderate
NAT2 poor metabolizer (PM)	Predicted to have increased hydralazine plasma concentrations compared to NAT2 rapid and intermediate metabolizers, which may lead to both increased efficacy and adverse effects, including drug‐induced systemic lupus erythematosus	Initiate therapy at a total daily dose of 40–75 mg. Carefully titrate dose upward to clinical effect or guideline‐recommended dose; use caution with total daily hydralazine doses of 200 mg or more	Moderate
NAT2 indeterminate	n/a	No recommendation	No recommendation

^a^ The online ***NAT2* Diplotype–Phenotype Table** provides a complete list of possible diplotypes and phenotype assignments.^10^, ^11^

^b^ Rating scheme described in the **Supplementary Material** online.

Hydralazine is also used for afterload reduction in the treatment of congestive heart failure, but the writing group identified insufficient evidence to support pharmacogenomic guidance for heart failure. There is no evidence for NAT2 phenoconversion in heart failure, and BiDil is an effective therapy for heart failure with reduced ejection fraction.^1^ We found no evidence to suggest that a *NAT2* pharmacogenetic effect would differ in the setting of heart failure.

Regarding hydralazine toxicity, studies demonstrate that NAT2 PMs have a higher risk of adverse effects compared to NAT2 RMs and IMs. Multiple studies describe the higher risk of drug‐induced SLE for NAT2 PMs compared with NAT2 RMs or IMs. We summed the reported cases of hydralazine‐induced lupus across all identified publications. Among PMs, 15 of 132 reported subjects developed SLE, while two of 72 RMs and IMs developed SLE (Odds Ratio: 4.5, 95% CI: 1.0 to 20.2). However, some hydralazine‐induced lupus reports lack a denominator (i.e., total number of patients exposed to hydralazine); thus, the odds ratio presented should be interpreted with caution. Hydralazine has also been reported to be associated with Anti‐Neutrophil Cytoplasmic Antibody (ANCA)‐induced vasculitis, although the evidence does not presently support a *NAT2* genotype‐dependent effect.^45^

### Therapeutic recommendations

**Table** **2** summarizes the hydralazine oral dosing recommendations based on NAT2 phenotype in patients with resistant hypertension. All phenotypes were considered actionable for blood pressure management, with recommendations for PMs to limit total daily dose and RM/IMs requiring higher starting doses. Most phenotyping studies upon which these recommendations are based did not distinguish between NAT2 RMs and IMs; thus, the clinical recommendations for these two groups are identical. As additional genotyping data on RMs and IMs treated with hydralazine become available, these recommendations may be subject to revision in future updates of this guideline. For RMs and IMs, consider a higher hydralazine starting total daily dose of at least 75 mg and titrate up to 300 mg total daily hydralazine dose as tolerated. NAT2 RMs and IMs typically require a 50–100% higher maintenance dose compared to NAT2 PMs. RMs and IMs may require more frequent follow‐up for blood pressure assessment and dose titration given the lower exposure of the drug in these individuals. Further, some clinicians prescribe hydralazine twice‐daily off‐label to improve adherence. Based on pharmacokinetic profiles, a twice‐daily dosing practice should be avoided in RMs and IMs. To optimize drug exposure, prescribers should consider a four times daily dosing regimen in RMs or IMs based on patient willingness, although a thrice‐daily regimen is commonly prescribed for improved adherence.

In contrast, NAT2 PMs are predicted to have higher plasma hydralazine concentrations and should be initiated at a total daily dose of 40–75 mg, with careful titration up to the clinical effect or guideline‐recommended dose. In NAT2 PMs, caution should be used with total daily hydralazine doses of 200 mg or more due to the risk of drug‐induced SLE.

The management of primary resistant hypertension is complex, with guidelines available to support clinicians in optimizing a regimen from the best combination of three or more antihypertensive agents.^2^ Often, clinicians turn to hydralazine only after more common antihypertensive class options have been exhausted. These common drug classes include calcium channel blockers, diuretics, renin–angiotensin–aldosterone system antagonists, and beta‐blockers. There may be circumstances where it is appropriate to substitute an alternate antihypertensive for hydralazine due to insufficient effect in RM/IMs or to avoid ADEs in PMs. Alternative agents for resistant hypertension therapy include, but are not limited to, spironolactone, doxazosin, and clonidine. The therapeutic guidelines presented here are intended to support clinicians who have elected to prescribe hydralazine for the treatment of primary resistant hypertension. We do not specifically recommend hydralazine prioritization over another medication in either PMs or RM/IMs. If an individual’s blood pressure is well‐controlled on hydralazine, there is no indication to adjust dosing, unless they are a PM on a total dose greater than 200 mg/day.

#### Pediatrics

Hydralazine is not commonly used in the pediatric patient population. The clinical data upon which this guideline is based were obtained from studies in adults. Given the well‐characterized pharmacokinetic basis for this gene–drug interaction and the presence of 90% of mature NAT2 enzyme activity after 4–5 months of age,^46^, ^47^ it is reasonable to extrapolate the recommendations presented in this guideline to pediatric patients prescribed oral hydralazine if needed.

#### Biogeographic groups

The NAT2 phenotype to genotype correlations are derived from populations of European, Asian, and African ancestry. The hydralazine‐specific studies are fewer in number, and future studies that link blood pressure and adverse events to genotype are needed in diverse ancestry groups. However, given the strong allele definitions from diverse populations, there is reason to suspect that the effects of *NAT2* genetic variation on hydralazine exposure and treatment outcomes will apply across biogeographic groups.

### Recommendations for incidental findings

Not applicable.

### Other considerations

Drug–drug and drug–disease interactions for hydralazine have been infrequently reported. Limited studies have suggested acetaminophen has a weak interaction with NAT2 substrates, reducing metabolite exposure 19 to 30% for the probe drug caffeine.^48^ Phenoconversion of NAT2 enzyme function in liver disease has not been studied *in vivo*. Thus, presently there is insufficient evidence supporting phenoconversion of the NAT2 metabolizer status based on concomitant drugs or comorbidities.

#### Implementation of this guideline

The guideline supplement and CPIC website (https://cpicpgx.org/guidelines/cpic‐guideline‐for‐hydralazine‐and‐nat2/) contains resources that can be used within electronic health records (EHRs) to assist clinicians in applying genetic information to patient care for the purpose of drug therapy optimization (see *Resources to incorporate pharmacogenetics into an electronic health record with clinical decision support* in the **Supplemental Material**).

## POTENTIAL BENEFITS AND RISKS FOR THE PATIENT

The potential benefits of using *NAT2* genotype‐informed phenotype to guide hydralazine therapy are to achieve effective blood pressure reduction in the setting of resistant hypertension and minimize the incidence of drug‐induced SLE. As with any laboratory test, a possible risk to patients is an error in genotyping or phenotype prediction, which could have long‐term adverse health implications for patients.

## CAVEATS: APPROPRIATE USE AND/OR POTENTIAL MISUSE OF GENETIC TESTS

There are some important limitations to *NAT2* genetic tests. Targeted genotyping tests focus on interrogating previously described star (*) alleles and therefore are not designed to detect novel variants. Furthermore, rare allelic *NAT2* variants may not be included in the genotype test used, and patients with these rare variants may be assigned a RM phenotype (NAT2*1/*1) by default. As such, an assigned NAT2*1 allele could potentially harbor an undetected genetic variant that results in altered metabolism and drug exposure. Therefore, it is important that clinicians appreciate the limitations of targeted genotyping tests and understand which *NAT2* variant alleles were genotyped when interpreting results. As with any diagnostic test, *NAT2* genotype is just one factor that clinicians should consider when prescribing hydralazine. Given the extensive update of *NAT2* nomenclature, previously documented *NAT2* results may need to be adjusted based on this updated guidance.

## FUNDING

This work was funded by the National Institutes of Health (NIH) for CPIC (U24HG013077) and PharmGKB (U24HG010615) and supported in part by ALSAC. PharmVar is supported by the Children’s Mercy Research Institute (CMRI). DWH is supported by NIH grants T32‐ES011564, P30‐ES030283, P42‐ES023716, and P20‐GM113226. SEK is supported by a National Cancer Institute grant K08‐CA250418. ZD is supported by NIGMS grant R3‐5GM145383. MTE is supported by the NCCIH grant R01‐AT011463. NAL is supported by a NHLBI grant K24HL133373. JDD is supported by NHGRI grants R01HG011800 and R01HG013416. The content is solely the responsibility of the authors and does not necessarily represent the official views of the National Institutes of Health.

## CONFLICT OF INTEREST

The authors declared no competing interests for this work.

## DISCLAIMER

Clinical Pharmacogenetics Implementation Consortium (CPIC) guidelines reflect expert consensus based on clinical evidence and peer‐reviewed literature available at the time they are written and are intended only to assist clinicians in decision making, as well as to identify questions for further research. New evidence may have emerged since the time a guideline was submitted for publication. Guidelines are limited in scope and are not applicable to interventions or diseases not specifically identified. Guidelines do not account for all individual variation among patients and cannot be considered inclusive of all proper methods of care or exclusive of other treatments. It remains the responsibility of the healthcare provider to determine the best course of treatment for the patient. Adherence to any guideline is voluntary, with the ultimate determination regarding its application to be solely made by the clinician and the patient. CPIC assumes no responsibility for any injury to persons or damage to property related to any use of CPIC’s guidelines, or for any errors or omissions.

## Supporting information


Data S1.

Data S2.
