# Supplementary material for: Clinical Pharmacogenetics Implementation Consortium Guideline for NAT2 Genotype and Hydralazine Therapy
Source: Clin Pharmacol Ther. 2025 Sep 19;118(6):1430–6. doi: 10.1002/cpt.70071 (PMC12641075; doi:10.1002/cpt.70071)
Supplement: Supplementary file 1 — Data S1. [file CPT-118-1430-s001.docx]

**Supplement to:**

**Clinical Pharmacogenetics Implementation Consortium Guideline for *NAT2* Genotype and Hydralazine Therapy**

Michael T. Eadon^1^, David W. Hein^2^, Michael A. Andersen^3^, Arlene B. Chapman^4^, Rhonda M. Cooper-DeHoff^5^, Zeruesenay Desta^1^, Julio D. Duarte^5^, Amanda L. Elchynski^6^, Andrea Gaedigk^7^, Seth E. Karol^8^, Nita A. Limdi^9^, Christelle Lteif^5^, Laura Sosinski^10^, Pablo Zubiaur^11^, Michelle Whirl-Carrillo^12^, Teri E. Klein^12^, Kelly E. Caudle^13^, Roseann S. Donnelly^13,14^, José A.G. Agúndez^15^

^1^Division of Clinical Pharmacology, Indiana University School of Medicine, Indianapolis, Indiana, USA

^2^Department of Pharmacology and Toxicology, University of Louisville School of Medicine, Louisville, Kentucky, USA

^3^Department of Clinical Pharmacology, Copenhagen University Hospital – Bispebjerg and Frederiksberg, Copenhagen, Denmark

^4^Department of Medicine, University of Chicago, Chicago, Illinois, USA

^5^Department of Pharmacotherapy and Translational Research and Center for Pharmacogenomics and Precision Medicine, University of Florida College of Pharmacy, Gainesville, Florida, USA

^6^Department of Pharmacy, Arkansas Children’s Hospital, Little Rock, Arkansas, USA

^7^Division of Clinical Pharmacology, Toxicology & Therapeutic Innovation, Children’s Mercy Research Institute and School of Medicine, University of Missouri-Kansas City, Kansas City, Missouri, USA

^8^Department of Oncology, St. Jude Children’s Research Hospital, Memphis, Tennessee, USA

^9^Department of Neurology, Heersink School of Medicine, University of Alabama at Birmingham, Birmingham, Alabama, USA

^10^Deparment of Pharmacy Practice, Butler University College of Pharmacy and Health Sciences, Indianapolis, Indiana, USA

^11^Department of Clinical Pharmacology, Hospital Universitario de la Princesa, Instituto Teófilo Hernando, Universidad Autónoma de Madrid (UAM), Instituto de Investigación Sanitaria La Princesa (IP), Madrid, Spain

^12^Department of Biomedical Data Science, Stanford University, Stanford, California, USA

^13^Department of Pharmacy and Pharmaceutical Sciences, St. Jude Children’s Research Hospital, Memphis, Tennessee, USA

^14^Department of Pharmacy Practice, Massachusetts College of Pharmacy and Health Sciences, Boston, Massachusetts, USA

^15^Institute of Molecular Pathology Biomarkers, Universidad de Extremadura, Cáceres, Spain

**TABLE OF CONTENTS**

[**GUIDELINE UPDATES** 3](#_Toc203630680)

[**LITERATURE REVIEW** 3](#_Toc203630681)

[**GENETIC TEST INTERPRETATION** 3](#_Toc203630682)

[**AVAILABLE GENETIC TEST OPTIONS** 4](#_Toc203630683)

[**LEVELS OF EVIDENCE LINKING GENOTYPE TO PHENOTYPE** 5](#_Toc203630684)

[**STRENGTH OF RECOMMENDATIONS** 5](#_Toc203630685)

[**RESOURCES TO INCORPORATE PHARMACOGENOMICS INTO AN ELECTRONIC HEALTH RECORD WITH CLINICAL DECISION SUPPORT** 6](#_Toc203630686)

[**TABLE S1.** EVIDENCE LINKING NAT2 TO HYDRALAZINE PHENOTYPE 9](#_Toc203630687)

[**FIGURE S1.** HEPATIC METABOLISM OF HYDRALAZINE 14](#_Toc203630688)

[**REFERENCES** 15](#_Toc203630689)

# **GUIDELINE UPDATES**

The Clinical Pharmacogenetics Implementation Consortium (CPIC) guideline for *NAT2* and hydralazine therapy is published in full on the CPIC website (1). Relevant information will be reviewed periodically, and updated guidelines published online (1).

# **LITERATURE REVIEW**

The PubMed® database (1966 to January 2024) was searched for the following keywords: hydralazine AND (NAT2 OR n-acetyltransferase OR acetylator). The search was limited to studies conducted in humans and written in the English language, and review articles were excluded. Using these search terms, 126 publications were identified for review. Study inclusion criteria included publications that incorporated analyses for the association between *NAT2* genotype or phenotype and hydralazine pharmacokinetic parameters or hydralazine-related clinical outcomes in patients. Following the application of these criteria, 50 publications were reviewed and included in the evidence tables (**Table S1**). Two additional studies identified in the reference list of a recent review article (2) but not found in the PubMed search described above were also included. This review article also included an assessment of *NAT2* genotype to phenotype concordance using 29 additional studies, which were included as part of the original evidence table.

# **GENETIC TEST INTERPRETATION**

Haplotypes, or star (*) alleles, are determined by a specific single nucleotide variation (SNV) or a combination of SNVs that are interrogated in the genotyping analysis. The genotypes that constitute the haplotypes, or star (*) alleles for *NAT2*, and the rsIDs for each of the specific nucleotide alterations that define the alleles, are as defined on the PharmVar *NAT* gene page (<https://www.pharmvar.org/gene/NAT2>) from which the ***NAT2* Allele Definition Table** online is sourced (1, 3). The genotype results are generally reported as a diplotype, which includes one maternal and one paternal allele (e.g., *NAT2*4/*5*). The clinical functional assignments of *NAT2* alleles are summarized in the ***NAT2* Allele Functionality Table** online (1, 3).

Star allele-based *NAT2* nomenclature was transitioned to PharmVar in March 2024 during which numerous changes have been made. We refer the reader to the PharmVar GeneFocus on *NAT2* for details (in preparation), as well as documentation available on the PharmVar *NAT2* gene page ([www.pharmvar.org/gene/NAT2](http://www.pharmvar.org/gene/NAT2)) including the Read Me and Change Log documents and the Look-Up table which crosswalks between the ‘old’ and the ‘new’ PharmVar nomenclature.

# **AVAILABLE GENETIC TEST OPTIONS**

Commercially available genetic testing options change over time. The Genetic Testing Registry provides a central location for voluntary submission of genetic test information by providers and is available at <http://www.ncbi.nlm.nih.gov/gtr>. Desirable characteristics of pharmacogenomic tests, including naming of alleles and test report contents, have been extensively reviewed by an international group, including CPIC members (4). CPIC recommends that clinical laboratories adhere to these test reporting standards. CPIC gene-specific tables adhere to these allele nomenclature standards. Moreover, these tables (e.g., ***NAT2* Allele Definition Table**, ***NAT2* Allele Functionality Table**) may be used to assemble lists of known functional and actionable genetic variants (1, 3).

# **LEVELS OF EVIDENCE LINKING GENOTYPE TO PHENOTYPE**

The evidence summarized in **Table S1** is graded on a scale of high, moderate, and weak based upon the level of evidence:

- **High:** Evidence includes consistent results from well-designed, well-conducted studies. High confidence that the available evidence reflects the true magnitude and direction of the net effect and further research is very unlikely to change the magnitude or direction of this net effect.
- **Moderate:** Evidence is sufficient to determine effects, but the strength of the evidence is limited by the number, quality, or consistency of the individual studies; generalizability to routine practice; or indirect nature of the evidence. Further research is unlikely to alter the direction of the net effect, however it might alter the magnitude of the net effect.
- **Weak:** Evidence is insufficient to assess the effects on health outcomes because of limited number or power of studies, important flaws in their design or conduct, gaps in the chain of evidence, or lack of information. Further research may change the magnitude and/or direction of the net effect.

# **STRENGTH OF RECOMMENDATIONS**

CPIC’s therapeutic recommendations are based on weighing the evidence from a combination of preclinical functional and clinical data, as well as on some existing disease-specific consensus guidelines. Some of the factors that are taken into account in evaluating the evidence supporting therapeutic recommendations may include *in vivo* pharmacokinetic and pharmacodynamic data, *in vitro* enzyme activity of tissues expressing wild-type/reference or variant-containing enzyme, *in vitro* enzyme activity from tissues isolated from individuals of known genotypes, and *in vivo* pre-clinical and clinical pharmacokinetic and pharmacodynamic studies.

Overall, the therapeutic recommendations are simplified to allow rapid interpretation by clinicians. CPIC uses a slight modification of a transparent and simple system for recommendations adopted from the rating scale for evidence-based guidelines on the use of antiretroviral agents (5):

- **Strong** recommendation for the statement: The evidence is high quality and the desirable effects clearly outweigh the undesirable effects.
- **Moderate** recommendation for the statement: There is a close or uncertain balance as to whether the evidence is high quality and the desirable effects clearly outweigh the undesirable effects.
- **Optional** recommendation for the statement: The desirable effects are closely balanced with undesirable effects, or the evidence is weak or based on extrapolations. There is room for differences in opinion as to the need for the recommended course of action.
- **No recommendation**: There is insufficient evidence, confidence, or agreement to provide a recommendation to guide clinical practice at this time.

# **RESOURCES TO INCORPORATE PHARMACOGENOMICS INTO AN ELECTRONIC HEALTH RECORD WITH CLINICAL DECISION SUPPORT**

Clinical decision support (CDS) tools integrated within electronic health records (EHRs) can help guide clinical pharmacogenomics at the point of care (6-8). See <https://cpicpgx.org/guidelines/cpic-guideline-for-hydralazine-and-nat2/> for resources to support the adoption of CPIC guidelines within an EHR (1). Based on the capabilities of various EHRs and local preferences, we recognize that approaches may vary across organizations. Our intent is to synthesize foundational knowledge that provides a common starting point for incorporating *NAT2* genotype results in an EHR to guide hydralazine therapy.

Effective incorporation of pharmacogenomic information into an EHR to optimize drug therapy should have some key attributes. Pharmacogenomic test results, an interpreted phenotype, and a concise interpretation or summary of the result must be documented in the EHR. To incorporate a phenotype in the EHR in a standardized manner, genotype test results provided by the laboratory must be consistently translated into an interpreted drug metabolism phenotype (**Table 1, main manuscript; *NAT2* Diplotype to Phenotype Table** (1, 3)). Because clinicians must be able to easily find the information, the interpreted phenotype may be documented as a problem list entry or in a patient’s summary section; these phenotypes are best stored in the EHR at the “person level” rather than at the date-centric “encounter level”. Additionally, results should be entered as standardized and discrete terms to facilitate using them to provide point-of-care CDS (see **Hydralazine Pre- and Post-Test Alerts and Flow Chart** for example CDS alerts; <https://cpicpgx.org/guidelines/cpic-guideline-for-hydralazine-and-nat2/>) (1). Point-of-care CDS should be designed to effectively notify clinicians of prescribing implications at any time after the test result is entered into the EHR. For this guideline, each NAT2 phenotype (rapid metabolizer [RM], intermediate metabolizer [IM], poor metabolizer [PM]) is considered an actionable (“priority/high risk”) result, depending on the hydralazine dose prescribed. For RMs and IMs, a post-test alert is recommended when a total daily dose of hydralazine ≤ 50 mg is prescribed. For PMs, a post-test alert is recommended when a total daily dose of hydralazine ≥200 mg is prescribed. CPIC’s informatics resources are meant to be used as a starting point, and each institution is encouraged to customize their approach (e.g., what type of alerts to deploy, what wording to use) based on their unique needs.

Because pharmacogenomic test results have lifetime implications and clinical significance, results should be placed into a section of the EHR that is accessible independent of the test result date to allow clinicians to quickly find the result at any time after it is initially placed in the EHR. To facilitate this process, CPIC is providing gene-specific information figures and tables that include complete diplotype to phenotype translation tables, diagram(s) that illustrate how *NAT2* pharmacogenomic test results could be entered into an EHR, example EHR consultation/genetic test interpretation language and widely used nomenclature systems for relevant drugs (see <https://cpicpgx.org/guidelines/cpic-guideline-for-hydralazine-and-nat2/>) (1).

# **TABLE S1.** EVIDENCE LINKING NAT2 TO HYDRALAZINE PHENOTYPE

| **Type of Experimental Model (*in vitro*, *in vivo*, preclinical, or clinical)** | **Major Findings** | **References** | **Level of Evidence^a^** |
| --- | --- | --- | --- |
| *In vitro* | In human hepatocytes, MTP production from hydralazine metabolism was higher in NAT2 rapid versus intermediate versus poor metabolizers at different hydralazine concentrations. | Allen, *et al.* (2017) (9) | High |
| Clinical | The overall concordance of *NAT2* genotype to NAT2 enzymatic activity phenotype is greater than 90%. | Deguchi, *et al.* (1990) (10)  Hickman, *et al.* (1991) (11)  Graf, *et al.* (1992) (12)  Bell, *et al.* (1993) (13)  Cascorbi, *et al.* (1995) (14)  Le Marchand, *et al.* (1996) (15)  Kaufmann, *et al.* (1996) (16)  O'Neil, *et al.* (1997) (17)  Parkin, *et al.* (1997) (18)  Smith, *et al.* (1997) (19)  Woolhouse, *et al.* (1997) (20)  Cascorbi, *et al.* (1999) (21)  Gross, *et al.* (1999) (22)  Wolkenstein, *et al.* (2000) (23)  Zhao, *et al.* (2000) (24)  Bolt, *et al.* (2005) (25)  Skretkowicz, *et al.* (2005) (26)  Goldenkova-Pavlova, *et al.* (2006) (27)  Rychlik-Sych, *et al.* (2006) (28)  Straka, *et al.* (2006) (29)  Rihs, *et al.* (2007) (30)  Díaz-Molina, *et al.* (2008) (31)  Kuhn, *et al.* (2010) (32)  Hein, *et al.* (2012) (33)  Rana, *et al.* (2012) (34)  Ruiz, *et al.* (2012) (35)  Al-Ahmad, *et al.* (2017) (36)  Aklilu, *et al.* (2018) (37)  Birch, *et al.* (2018) (38)  Akhter, *et al.* (2019) (39) | High |
| Clinical | NAT2 poor metabolizers (slow acetylators) have higher hydralazine exposure compared to NAT2 rapid and intermediate metabolizers (rapid acetylators). | Zacest, *et al.* (1972) (40)  Jounela, *et al.* (1975) (41)  Talseth, *et al.* (1977) (42)  Timbrell, *et al.* (1979) (43)  Hawksworth, *et al.* (1980) (44)  Reece, *et al.* (1980) (45)  Shen, *et al.* (1980) (46)  Shepherd, *et al.* (1980) (47)  Timbrell, *et al.* (1980) (48)  Facchini, *et al.* (1981) (49)  Ludden, *et al.* (1981) (50)  Timbrell, *et al.* (1981) (51)  Ludden, *et al.* (1983) (52)  Timbrell, *et al.* (1984) (53)  Blair, *et al.* (1985) (54)  Dubois, *et al.* (1987) (55)  Rashid, *et al.* (1992) (56)  Gonzalez-Fierro, *et al.* (2011) (57)  Han, *et al.* (2019) (58) | High |
| Clinical | Hydralazine dosed at 182 mg in NAT2 rapid and intermediate metabolizers (rapid acetylators) and 83 mg in NAT2 poor metabolizers (slow acetylators) resulted in similar hydralazine exposure. | Arce, *et al.* (2006) (59)  Candelaria, *et al.* (2007) (60)  Coronel, *et al.* (2011) (61)  Gonzalez-Fierro, *et al.* (2011) (57)  Garcés-Eisele, *et al.* (2014) (62) | High |
| Clinical | Hydralazine is a more effective antihypertensive in NAT2 poor metabolizers (slow acetylators) compared to NAT2 rapid and intermediate metabolizers (rapid acetylators) at the same dose. | Hunyor, *et al.* (1975) (63)  Jounela, *et al.* (1975) (41)  Kalowski, *et al.* (1979) (64)  Vidrio, *et al.* (1980) (65)  Wulff, *et al.* (1980) (66)  Shepherd, *et al.* (1981) (67)  Shepherd, *et al.* (1981) (68)  Silas, *et al.* (1982) (69)  Vandenburg, *et al.* (1982) (70)  Danielson, *et al.* (1983) (71)  Koopmans, *et al.* (1984) (72)  Ramsay, *et al.* (1984) (73)  Rowell, *et al.* (1990) (74)  Spinasse, *et al.* (2014) (75) | Moderate |
| Clinical | A higher dose of hydralazine is required in NAT2 rapid and intermediate metabolizers (rapid acetylators) to achieve antihypertensive efficacy equivalent to NAT2 poor metabolizers (slow acetylators). | Zacest, *et al.* (1972) (40)  Hunyor, *et al.* (1975) (63)  Jounela, *et al.* (1975) (41)  Litwin, et al (1981) (76)  Silas, *et al.* (1982) (69)  Vandenburg, *et al.* (1982) (70)  Koopmans, *et al.* (1984) (72)  Ramsay, *et al.* (1984) (73)  Graves, *et al.* (1990) (77) | Moderate |
| Clinical | Hydralazine is a more effective antihypertensive in NAT2 poor metabolizers (slow acetylators) compared to NAT2 rapid and intermediate metabolizers (rapid acetylators). | Zacest, *et al.* (1972) (40)  Hunyor, *et al.* (1975) (63)  Jounela, *et al.* (1975) (41)  Kalowski, *et al.* (1979) (64)  Wulff, *et al.* (1980) (66)  Litwin, *et al.* (1981) (76)  Shepherd, *et al.* (1981) (67)  Shepherd, *et al.* (1981) (68)  Silas, *et al.* (1982) (69)  Vandenburg, *et al.* (1982) (70)  Danielson, *et al.* (1983) (71)  Koopmans, *et al.* (1984) (72)  Ramsay, *et al.* (1984) (73)  Graves, *et al.* (1990) (77)  Rowell, *et al.* (1990) (74)  Spinasse, *et al.* (2014) (75) | Moderate |
| Clinical | NAT2 poor metabolizers (slow acetylators) have a higher risk of non-lupus adverse effects with hydralazine compared to NAT2 rapid and intermediate metabolizers (rapid acetylators). | Kalowski, *et al.* (1979) (64)  Wulff, *et al.* (1980) (66)  Tsujimoto, *et al.* (1981) (78)  Vandenburg, *et al.* (1982) (70)  Dahlqvist, *et al.* (1983) (79)  Danielson, *et al.* (1983) (71)  Ramsay, *et al.* (1984) (73)  Björck, *et al.* (1985) (80)  Gonzalez-Fierro, *et al.* (2011) (57)  Spinasse, *et al.* (2014) (75) | Moderate |
| Clinical | NAT2 poor metabolizers (slow acetylators) have a higher risk of developing hydralazine-induced systemic lupus erythematosus compared to NAT2 rapid and intermediate metabolizers (rapid acetylators). | Hunyor, *et al.* (1975) (63)  Strandberg, *et al.* (1976) (81)  Batchelor, *et al.* (1980) (82)  Litwin, *et al.* (1981) (76)  Cameron, *et al.* (1984) (83)  Ihle, *et al.* (1984) (84)  Ramsay, *et al.* (1984) (73)  Timbrell, *et al.* (1984) (53)  Asherson, *et al.* (1986) (85)  Russell, *et al.* (1986) (86)  Pålsson, *et al.* (1989) (87)  Schattner, *et al.* (1994) (88)  Spinasse, *et al.* (2014) (75)  Holman, *et al.* (2017) (89) | Moderate |
| Clinical | NAT2 poor metabolizers (slow acetylators) have a higher risk of adverse effects with hydralazine compared to NAT2 rapid and intermediate metabolizers (rapid acetylators). | Hunyor, *et al.* (1975) (63)  Strandberg, *et al.* (1976) (81)  Kalowski, *et al.* (1979) (64)  Batchelor, *et al.* (1980) (82)  Wulff, *et al.* (1980) (66)  Litwin, *et al.* (1981) (76)  Tsujimoto, *et al.* (1981) (78)  Vandenburg, *et al.* (1982) (70)  Dahlqvist, *et al.* (1983) (79)  Danielson, *et al.* (1983) (71)  Cameron, *et al.* (1984) (83)  Ihle, *et al.* (1984) (84)  Ramsay, *et al.* (1984) (73)  Timbrell, *et al.* (1984) (53)  Björck, *et al.* (1985) (80)  Asherson, *et al.* (1986) (85)  Russell, *et al.* (1987) (86)  Pålsson, *et al.* (1989) (87)  Schattner, *et al.* (1994) (88)  Gonzalez-Fierro, *et al.* (2011) (57)  Spinasse, *et al.* (2014) (75)  Holman, *et al.* (2017) (89) | Moderate |

^a^Rating scheme described in the **Supplemental Material**


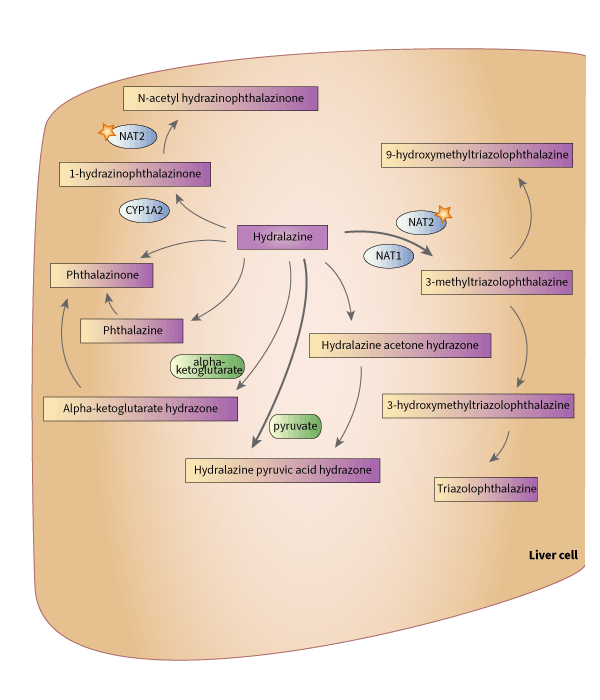


# **FIGURE S1.** HEPATIC METABOLISM OF HYDRALAZINE

For a detailed and updated description, please see: <https://www.pharmgkb.org/pathway/PA166271241>. Image is available under a Creative Commons BY-SA 4.0 license (90).

# **REFERENCES**

(1) *CPIC® Guideline for Hydralazine and NAT2*. <<https://cpicpgx.org/guidelines/cpic-guideline-for-hydralazine-and-nat2/>>. Accessed 20 May 2025.

(2) Collins, K.S. *et al.* Genotype-Guided Hydralazine Therapy. *Am J Nephrol* **51**, 764-76 (2020).

(3) *Gene-specific Information Tables for NAT2*. <<https://www.pharmgkb.org/page/nat2RefMaterials>>. Accessed 10 June 2025.

(4) Kalman, L.V. *et al.* Pharmacogenetic allele nomenclature: International workgroup recommendations for test result reporting. *Clin Pharmacol Ther* **99**, 172-85 (2016).

(5) *Guidelines for the Use of Antiretroviral Agents in HIV-1-Infected Adults and Adolescents.* <<https://clinicalinfo.hiv.gov/en/guidelines/adult-and-adolescent-arv/whats-new-guidelines>>. Accessed 20 May 2025.

(6) Hicks, J.K., Dunnenberger, H.M., Gumpper, K.F., Haidar, C.E. & Hoffman, J.M. Integrating pharmacogenomics into electronic health records with clinical decision support. *Am J Health Syst Pharm* **73**, 1967-76 (2016).

(7) Hoffman, J.M. *et al.* Developing knowledge resources to support precision medicine: principles from the Clinical Pharmacogenetics Implementation Consortium (CPIC). *J Am Med Inform Assoc* **23**, 796-801 (2016).

(8) Liu, M. *et al.* A Tutorial for Pharmacogenomics Implementation Through End-to-End Clinical Decision Support Based on Ten Years of Experience from PREDICT. *Clin Pharmacol Ther* **109**, 101-15 (2021).

(9) Allen, C.E., Doll, M.A. & Hein, D.W. N-Acetyltransferase 2 Genotype-Dependent N-Acetylation of Hydralazine in Human Hepatocytes. *Drug Metab Dispos* **45**, 1276-81 (2017).

(10) Deguchi, T., Mashimo, M. & Suzuki, T. Correlation between acetylator phenotypes and genotypes of polymorphic arylamine N-acetyltransferase in human liver. *J Biol Chem* **265**, 12757-60 (1990).

(11) Hickman, D. & Sim, E. N-acetyltransferase polymorphism. Comparison of phenotype and genotype in humans. *Biochem Pharmacol* **42**, 1007-14 (1991).

(12) Graf, T., Broly, F., Hoffmann, F., Probst, M., Meyer, U.A. & Howald, H. Prediction of phenotype for acetylation and for debrisoquine hydroxylation by DNA-tests in healthy human volunteers. *Eur J Clin Pharmacol* **43**, 399-403 (1992).

(13) Bell, D.A. *et al.* Genotype/phenotype discordance for human arylamine N-acetyltransferase (NAT2) reveals a new slow-acetylator allele common in African-Americans. *Carcinogenesis* **14**, 1689-92 (1993).

(14) Cascorbi, I., Drakoulis, N., Brockmoller, J., Maurer, A., Sperling, K. & Roots, I. Arylamine N-acetyltransferase (NAT2) mutations and their allelic linkage in unrelated Caucasian individuals: correlation with phenotypic activity. *Am J Hum Genet* **57**, 581-92 (1995).

(15) Le Marchand, L. *et al.* Predictors of N-acetyltransferase activity: should caffeine phenotyping and NAT2 genotyping be used interchangeably in epidemiological studies? *Cancer Epidemiol Biomarkers Prev* **5**, 449-55 (1996).

(16) Kaufmann, G.R. *et al.* N-acetyltransferase 2 polymorphism in patients infected with human immunodeficiency virus. *Clin Pharmacol Ther* **60**, 62-7 (1996).

(17) O'Neil, W.M., Gilfix, B.M., DiGirolamo, A., Tsoukas, C.M. & Wainer, I.W. N-acetylation among HIV-positive patients and patients with AIDS: when is fast, fast and slow, slow? *Clin Pharmacol Ther* **62**, 261-71 (1997).

(18) Parkin, D.P. *et al.* Trimodality of isoniazid elimination: phenotype and genotype in patients with tuberculosis. *Am J Respir Crit Care Med* **155**, 1717-22 (1997).

(19) Smith, C.A., Wadelius, M., Gough, A.C., Harrison, D.J., Wolf, C.R. & Rane, A. A simplified assay for the arylamine N-acetyltransferase 2 polymorphism validated by phenotyping with isoniazid. *J Med Genet* **34**, 758-60 (1997).

(20) Woolhouse, N.M., Qureshi, M.M., Bastaki, S.M., Patel, M., Abdulrazzaq, Y. & Bayoumi, R.A. Polymorphic N-acetyltransferase (NAT2) genotyping of Emiratis. *Pharmacogenetics* **7**, 73-82 (1997).

(21) Cascorbi, I., Brockmoller, J., Mrozikiewicz, P.M., Muller, A. & Roots, I. Arylamine N-acetyltransferase activity in man. *Drug Metab Rev* **31**, 489-502 (1999).

(22) Gross, M. *et al.* Distribution and concordance of N-acetyltransferase genotype and phenotype in an American population. *Cancer Epidemiol Biomarkers Prev* **8**, 683-92 (1999).

(23) Wolkenstein, P., Loriot, M.A., Aractingi, S., Cabelguenne, A., Beaune, P. & Chosidow, O. Prospective evaluation of detoxification pathways as markers of cutaneous adverse reactions to sulphonamides in AIDS. *Pharmacogenetics* **10**, 821-8 (2000).

(24) Zhao, B., Seow, A., Lee, E.J. & Lee, H.P. Correlation between acetylation phenotype and genotype in Chinese women. *Eur J Clin Pharmacol* **56**, 689-92 (2000).

(25) Bolt, H.M., Selinski, S., Dannappel, D., Blaszkewicz, M. & Golka, K. Re-investigation of the concordance of human NAT2 phenotypes and genotypes. *Arch Toxicol* **79**, 196-200 (2005).

(26) Skretkowicz, K., Skretkowicz, J., Gawronska-Szklarz, B., Gornik, W., Rychlik-Sych, M. & Sysa-Jedrzejowska, A. Lack of association between arylamine N-acetyltransferase 2 (NAT2) polymorphism and systemic sclerosis. *Eur J Clin Pharmacol* **60**, 773-8 (2005).

(27) Goldenkova-Pavlova, I.V. *et al.* [Comparative analysis of N-acetylation polymorphism in humans as determined by phenotyping and genotyping]. *Genetika* **42**, 1143-50 (2006).

(28) Rychlik-Sych, M., Skretkowicz, J., Gawronska-Szklarz, B., Gornik, W., Sysa-Jedrzejowska, A. & Skretkowicz-Szarmach, K. Acetylation genotype and phenotype in patients with systemic lupus erythematosus. *Pharmacol Rep* **58**, 22-9 (2006).

(29) Straka, R.J., Burkhardt, R.T., Lang, N.P., Hadsall, K.Z. & Tsai, M.Y. Discordance between N-acetyltransferase 2 phenotype and genotype in a population of Hmong subjects. *J Clin Pharmacol* **46**, 802-11 (2006).

(30) Rihs, H.P., John, A., Scherenberg, M., Seidel, A. & Bruning, T. Concordance between the deduced acetylation status generated by high-speed: real-time PCR based NAT2 genotyping of seven single nucleotide polymorphisms and human NAT2 phenotypes determined by a caffeine assay. *Clin Chim Acta* **376**, 240-3 (2007).

(31) Diaz-Molina, R. *et al.* Genotype and phenotype of NAT2 and the occurrence of adverse drug reactions in Mexican individuals to an isoniazid-based prophylactic chemotherapy for tuberculosis. *Mol Med Rep* **1**, 875-9 (2008).

(32) Kuhn, U.D., Anschutz, M., Schmucker, K., Schug, B.S., Hippius, M. & Blume, H.H. Phenotyping with sulfasalazine - time dependence and relation to NAT2 pharmacogenetics. *Int J Clin Pharmacol Ther* **48**, 1-10 (2010).

(33) Hein, D.W. & Doll, M.A. Accuracy of various human NAT2 SNP genotyping panels to infer rapid, intermediate and slow acetylator phenotypes. *Pharmacogenomics* **13**, 31-41 (2012).

(34) Rana, S.V. *et al.* Comparison between acetylator phenotype and genotype polymorphism of n-acetyltransferase-2 in tuberculosis patients. *Hepatol Int* **6**, 397-402 (2012).

(35) Ruiz, J.D. *et al.* The differential effect of NAT2 variant alleles permits refinement in phenotype inference and identifies a very slow acetylation genotype. *PLoS One* **7**, e44629 (2012).

(36) Al-Ahmad, M.M. *et al.* Studies on N-Acetyltransferase (NAT2) Genotype Relationships in Emiratis: Confirmation of the Existence of Phenotype Variation among Slow Acetylators. *Ann Hum Genet* **81**, 190-6 (2017).

(37) Aklillu, E., Carrillo, J.A., Makonnen, E., Bertilsson, L. & Djordjevic, N. N-Acetyltransferase-2 (NAT2) phenotype is influenced by genotype-environment interaction in Ethiopians. *Eur J Clin Pharmacol* **74**, 903-11 (2018).

(38) Birch Kristensen, E. *et al.* Study of correlation between the NAT2 phenotype and genotype status among Greenlandic Inuit. *EXCLI J* **17**, 1043-53 (2018).

(39) Akhter, N., Iqbal, T., Jamil, A., Akram, M., Mehmood Tahir, I. & Munir, N. Determination of Arylamine N-Acetyltransferase 2 Acetylation Genotype by PCR and Phenotyping Using Dapsone Through High-Pressure Liquid Chromatography Assay: A Gender Wise Study. *Dose Response* **17**, 1559325819855537 (2019).

(40) Zacest, R. & Koch-Weser, J. Relation of hydralazine plasma concentration to dosage and hypotensive action. *Clin Pharmacol Ther* **13**, 420-5 (1972).

(41) Jounela, A.J., Pasanen, M. & Mattila, M.J. Acetylator phenotype and the antihypertensive response to hydralazine. *Acta Med Scand* **197**, 303-6 (1975).

(42) Talseth, T. Kinetics of hydralazine elimination. *Clin Pharmacol Ther* **21**, 715-20 (1977).

(43) Timbrell, J.A. & Harland, S.J. Identification and quantitation of hydrazine in the urine of patients treated with hydralazine. *Clin Pharmacol Ther* **26**, 81-8 (1979).

(44) Hawksworth, G.M., Morrice, M., Petrie, J.C. & Scott, A.K. Bioavailability of standard and sugar-coated hydrallazine formulations in fast and slow acetylators [proceedings]. *Br J Clin Pharmacol* **9**, 111p-2p (1980).

(45) Reece, P.A., Cozamanis, I. & Zacest, R. Kinetics of hydralazine and its main metabolites in slow and fast acetylators. *Clin Pharmacol Ther* **28**, 769-78 (1980).

(46) Shen, D.D., Hosler, J.P., Schroder, R.L. & Azarnoff, D.L. Pharmacokinetics of hydralazine and its acid-labile hydrazone metabolites in relation to acetylator phenotype. *J Pharmacokinet Biopharm* **8**, 53-68 (1980).

(47) Shepherd, A.M., Ludden, T.M., McNay, J.L. & Lin, M.S. Hydralazine kinetics after single and repeated oral doses. *Clin Pharmacol Ther* **28**, 804-11 (1980).

(48) Timbrell, J.A., Harland, S.J. & Facchini, V. Polymorphic acetylation of hydralazine. *Clin Pharmacol Ther* **28**, 350-5 (1980).

(49) Facchini, V. & Timbrell, J.A. Further evidence for an acetylator phenotype difference in the metabolism of hydralazine in man. *Br J Clin Pharmacol* **11**, 345-51 (1981).

(50) Ludden, T.M., McNay, J.L., Jr., Shepherd, A.M. & Lin, M.S. Variability of plasma hydralazine concentrations in male hypertensive patients. *Arthritis Rheum* **24**, 987-93 (1981).

(51) Timbrell, J.A., Harland, S.J. & Facchini, V. Effect of dose on acetylator phenotype distribution of hydralazine. *Clin Pharmacol Ther* **29**, 337-43 (1981).

(52) Ludden, T.M., Shepherd, A.M., McNay, J.L., Jr. & Lin, M.S. Effect of intravenous dose on hydralazine kinetics after administration. *Clin Pharmacol Ther* **34**, 148-52 (1983).

(53) Timbrell, J.A., Facchini, V., Harland, S.J. & Mansilla-Tinoco, R. Hydralazine-induced lupus: is there a toxic metabolic pathway? *Eur J Clin Pharmacol* **27**, 555-9 (1984).

(54) Blair, I.A. *et al.* Plasma hydrazine concentrations in man after isoniazid and hydralazine administration. *Hum Toxicol* **4**, 195-202 (1985).

(55) Dubois, J.P., Schmid, K., Riess, W., Hanson, A., Henningsen, N.C. & Andersson, O.K. Metabolism of hydralazine in man. Part II: Investigation of features relevant to drug safety. *Arzneimittelforschung* **37**, 189-93 (1987).

(56) Rashid, J.R., Kofi, T. & Juma, F.D. Acetylation status using hydralazine in African hypertensives at Kenyatta National Hospital. *East Afr Med J* **69**, 406-8 (1992).

(57) Gonzalez-Fierro, A. *et al.* Pharmacokinetics of hydralazine, an antihypertensive and DNA-demethylating agent, using controlled-release formulations designed for use in dosing schedules based on the acetylator phenotype. *Int J Clin Pharmacol Ther* **49**, 519-24 (2011).

(58) Han, L.W. *et al.* Effect of N-Acetyltransferase 2 Genotype on the Pharmacokinetics of Hydralazine During Pregnancy. *J Clin Pharmacol* **59**, 1678-89 (2019).

(59) Arce, C. *et al.* A proof-of-principle study of epigenetic therapy added to neoadjuvant doxorubicin cyclophosphamide for locally advanced breast cancer. *PLoS One* **1**, e98 (2006).

(60) Candelaria, M. *et al.* A phase II study of epigenetic therapy with hydralazine and magnesium valproate to overcome chemotherapy resistance in refractory solid tumors. *Ann Oncol* **18**, 1529-38 (2007).

(61) Coronel, J. *et al.* A double-blind, placebo-controlled, randomized phase III trial of chemotherapy plus epigenetic therapy with hydralazine valproate for advanced cervical cancer. Preliminary results. *Med Oncol* **28 Suppl 1**, S540-6 (2011).

(62) Garcés-Eisele, S.J. *et al.* Genetic selection of volunteers and concomitant dose adjustment leads to comparable hydralazine/valproate exposure. *J Clin Pharm Ther* **39**, 368-75 (2014).

(63) Hunyor, S.N. Hydrallazine and beta-blockade in refractory hypertension with characterization of acetylator phenotype. *Aust N Z J Med* **5**, 530-6 (1975).

(64) Kalowski, S., Hua, A.S., Whitworth, J.A. & Kincaid-Smith, P. Hydrallazine with beta-blocker and diuretic in the treatment of hypertension. A double-blind crossover study. *Med J Aust* **2**, 439-40 (1979).

(65) Vidrio, H. & Tena, I. Hydralazine tachycardia and sympathetic cardiovascular reactivity in normal subjects. *Clin Pharmacol Ther* **28**, 587-91 (1980).

(66) Wulff, K., Lenz, K., Krogsgaard, A.R. & Holst, B. Hydralazine in arterial hypertension. Randomized double-blind comparison of conventional/Slow-Release formulation and of b.i.d./q.i.d. dosage regimens. *Acta Med Scand* **208**, 49-54 (1980).

(67) Shepherd, A., Lin, M.S., McNay, J., Ludden, T. & Musgrave, G. Determinants of response to intravenous hydralazine in hypertension. *Clin Pharmacol Ther* **30**, 773-81 (1981).

(68) Shepherd, A.M., McNay, J.L., Ludden, T.M., Lin, M.S. & Musgrave, G.E. Plasma concentration and acetylator phenotype determine response to oral hydralazine. *Hypertension* **3**, 580-5 (1981).

(69) Silas, J.H., Ramsay, L.E. & Freestone, S. Hydralazine once daily in hypertension. *Br Med J (Clin Res Ed)* **284**, 1602-4 (1982).

(70) Vandenburg, M.J., Wright, P., Holmes, J., Rogers, H.J. & Ahmad, R.A. The hypotensive response to hydralazine, in triple therapy, is not related to acetylator phenotype. *Br J Clin Pharmacol* **13**, 747-50 (1982).

(71) Danielson, M., Kjellberg, J., Ohman, P. & Wernersson, B. Evaluation of once daily hydralazine in inadequately controlled hypertension. *Acta Med Scand* **214**, 373-80 (1983).

(72) Koopmans, P.P., Hoefnagels, W.H., Huysmans, F.T. & Thien, T. Influence of acetylator phenotype and renal function on the antihypertensive effect of hydralazine. *Neth J Med* **27**, 69-73 (1984).

(73) Ramsay, L.E., Silas, J.H., Ollerenshaw, J.D., Tucker, G.T., Phillips, F.C. & Freestone, S. Should the acetylator phenotype be determined when prescribing hydralazine for hypertension? *Eur J Clin Pharmacol* **26**, 39-42 (1984).

(74) Rowell, N.P. & Clark, K. The effects of oral hydralazine on blood pressure, cardiac output and peripheral resistance with respect to dose, age and acetylator status. *Radiother Oncol* **18**, 293-8 (1990).

(75) Spinasse, L.B., Santos, A.R., Suffys, P.N., Muxfeldt, E.S. & Salles, G.F. Different phenotypes of the NAT2 gene influences hydralazine antihypertensive response in patients with resistant hypertension. *Pharmacogenomics* **15**, 169-78 (2014).

(76) Litwin, A., Adams, L.E., Zimmer, H., Foad, B., Loggie, J.H. & Hess, E.V. Prospective study of immunologic effects of hydralazine in hypertensive patients. *Clin Pharmacol Ther* **29**, 447-56 (1981).

(77) Graves, D.A., Muir, K.T., Richards, W., Steiger, B.W., Chang, I. & Patel, B. Hydralazine dose-response curve analysis. *J Pharmacokinet Biopharm* **18**, 279-91 (1990).

(78) Tsujimoto, G., Horai, Y., Ishizaki, T. & Itoh, K. Hydralazine-induced peripheral neuropathy seen in a Japanese slow acetylator patient. *Br J Clin Pharmacol* **11**, 622-5 (1981).

(79) Dahlqvist, A., Lundberg, E. & Ostberg, Y. Hydralazine-induced relapsing polychondritis-like syndrome. Report of a case with severe chronic laryngeal complications. *Acta Otolaryngol* **96**, 355-9 (1983).

(80) Björck, S., Svalander, C. & Westberg, G. Hydralazine-associated glomerulonephritis. *Acta Med Scand* **218**, 261-9 (1985).

(81) Strandberg, I., Boman, G., Hassler, L. & Sjöqvist, F. Acetylator phenotype in patients with hydralazine-induced lupoid syndrome. *Acta Med Scand* **200**, 367-71 (1976).

(82) Batchelor, J.R. *et al.* Hydralazine-induced systemic lupus erythematosus: influence of HLA-DR and sex on susceptibility. *Lancet* **1**, 1107-9 (1980).

(83) Cameron, H.A. & Ramsay, L.E. The lupus syndrome induced by hydralazine: a common complication with low dose treatment. *Br Med J (Clin Res Ed)* **289**, 410-2 (1984).

(84) Ihle, B.U., Whitworth, J.A., Dowling, J.P. & Kincaid-Smith, P. Hydralazine and lupus nephritis. *Clin Nephrol* **22**, 230-8 (1984).

(85) Asherson, R.A., Benbow, A.G., Speirs, C.J., Jackson, N. & Hughes, G.R. Pulmonary hypertension in hydralazine induced systemic lupus erythematosus: association with C4 null allele. *Ann Rheum Dis* **45**, 771-3 (1986).

(86) Russell, G.I., Bing, R.F., Jones, J.A., Thurston, H. & Swales, J.D. Hydralazine sensitivity: clinical features, autoantibody changes and HLA-DR phenotype. *Q J Med* **65**, 845-52 (1987).

(87) Pålsson, L., Weiner, L., Englund, G. & Henning, M. Cadralazine challenge in patients with previous hydralazine-induced lupus: a 6-month study. *Clin Pharmacol Ther* **46**, 177-81 (1989).

(88) Schattner, A., Sthoeger, Z. & Geltner, D. Effect of acute cytomegalovirus infection on drug-induced SLE. *Postgrad Med J* **70**, 738-40 (1994).

(89) Holman, S.K., Parris, D., Meyers, S. & Ramirez, J. Acute Low-Dose Hydralazine-Induced Lupus Pneumonitis. *Case Rep Pulmonol* **2017**, 2650142 (2017).

(90) *Hydralazine Pathway, Pharmacokinetics*. <<https://www.pharmgkb.org/pathway/PA166271241>>. Accessed 20 May 2025.
